# Negative dataset selection impacts machine learning-based predictors for multiple bacterial species promoters

**DOI:** 10.1093/bioinformatics/btaf135

**Published:** 2025-03-27

**Authors:** Marcelo González, Roberto E Durán, Michael Seeger, Mauricio Araya, Nicolás Jara

**Affiliations:** Departamento de Electrónica, Universidad Técnica Federico Santa María, Avenida España 1680, Valparaíso 2390123, Chile; Laboratorio de Microbiología Molecular y Biotecnología Ambiental, Department of Chemistry & Center of Biotechnology Daniel Alkalay Lowitt, Universidad Técnica Federico Santa María, Avenida España 1680, Valparaíso 2390123, Chile; Millennium Nucleus Bioproducts, Genomics and Environmental Microbiology (BioGEM), Avenida España 1680, Valparaíso 2390123, Chile; Laboratorio de Microbiología Molecular y Biotecnología Ambiental, Department of Chemistry & Center of Biotechnology Daniel Alkalay Lowitt, Universidad Técnica Federico Santa María, Avenida España 1680, Valparaíso 2390123, Chile; Millennium Nucleus Bioproducts, Genomics and Environmental Microbiology (BioGEM), Avenida España 1680, Valparaíso 2390123, Chile; Departamento de Electrónica, Universidad Técnica Federico Santa María, Avenida España 1680, Valparaíso 2390123, Chile; Departamento de Electrónica, Universidad Técnica Federico Santa María, Avenida España 1680, Valparaíso 2390123, Chile

## Abstract

**Motivation:**

Advances in bacterial promoter predictors based on machine learning have greatly improved identification metrics. However, existing models overlooked the impact of negative datasets, previously identified in GC-content discrepancies between positive and negative datasets in single-species models. This study aims to investigate whether multiple-species models for promoter classification are inherently biased due to the selection criteria of negative datasets. We further explore whether the generation of synthetic random sequences (SRS) that mimic GC-content distribution of promoters can partly reduce this bias.

**Results:**

Multiple-species predictors exhibited GC-content bias when using CDS as a negative dataset, suggested by specificity and sensibility metrics in a species-specific manner, and investigated by dimensionality reduction. We demonstrated a reduction in this bias by using the SRS dataset, with less detection of background noise in real genomic data. In both scenarios DNABERT showed the best metrics. These findings suggest that GC-balanced datasets can enhance the generalizability of promoter predictors across Bacteria.

**Availability and implementation:**

The source code of the experiments is freely available at https://github.com/maigonzalezh/MultispeciesPromoterClassifier.

## 1 Introduction

Bacterial promoters are essential DNA regions that regulate gene transcription initiation, facilitating specific binding to the DNA of the RNA polymerase (RNAP) holoenzyme (Eσ), a multi-subunit complex composed of the core RNAP and the σ factor (Mejía-Almonte *et al.* 2020). RNAP binding to promoter regions is improved by the σ factor, which recognizes cognate sequences with consensus motifs rich in AT (−35 and −10 elements) in comparison to the rest of the bacterial genome (Abril *et al.* 2020, Klein *et al.* 2021). Besides the housekeeping sigma factor ($\sigma_{70}$ in *Escherichia coli*, $\sigma_{A}$ in *Bacillus subtilis*), alternative σ factors act in response to environmental signals, leading the RNAP to other consensus sequences, changing the transcriptional profile to improve cellular fitness (e.g. heat-shock response, stationary phase regulation, nitrogen regulation, flagella) (Bervoets *et al.* 2018, Abril *et al.* 2020). Due to the great number of σ factors a single strain holds, from 1 to more than 100 (Gruber and Gross 2003, Lonetto *et al.* 2019), sequence patterns that suggest the presence of promoters generally vary between different σ factors and bacterial species, hindering identification efforts in bacterial genomics.

Advances in computational biology are promising for promoter identification in bacteria (Cassiano and Silva-Rocha 2020). Many promoter identification tools based on Machine Learning (ML) have been developed, mainly focusing on binary classification (promoter or non-promoter sequences) using *E. coli* or *B. subtilis* as model bacteria (Umarov and Solovyev 2017, Liu *et al.* 2018, Wenying *et al.* 2018, Rahman *et al.* 2019, Cassiano and Silva-Rocha 2020), or promoter classification ($\sigma_{70}$ or other subclasses) (Amin *et al.* 2020, Shujaat *et al.* 2020, Hernández *et al.* 2022). Different ML techniques have been applied to achieve this problem, including Random Forest (RF) in IPromoter-2, and Convolutional Neural Networks (CNNs) in iPromoter-BnCNN, pcPromoter-CNN, PromoterLCNN, and iProL (Amin *et al.* 2020, Shujaat *et al.* 2020, Hernández *et al.* 2022, Peng *et al.* 2024). Although these predictors are able to improve our understanding of σ promoters in model bacteria, bacterial diversity is much larger, requiring complex tools to identify promoters in multiple species. Chevez-Guardado and Peña-Castillo (2021) proposed Promotech, a multiple-species bacterial promoter predictor trained on datasets from nine species and evaluated on four additional species using a single model. Zhang *et al.* (2022) proposed iPro-WAEL, an ensemble learning model combining RF and CNN for promoter prediction trained with human sequences and evaluated across seven species (including Bacteria). TIMER, an approach based on siamese neural networks for bacterial promoter identification in general and species-specific models, was created using three siamese neural networks equipped with Attention layers across 13 species (Zhu *et al.* 2023). Although multiple species models are currently available, biases inherent to the addition of more than one species have not been assessed in ML models.

A significant area of research in deep learning is natural language processing (NLP), where the Transformer architecture has driven the development of large language models (LLMs) by leveraging the attention mechanism to capture relationships between distant elements and enable parallelized training. In this context, Bidirectional Encoder Representations from Transformers (BERT) introduced bidirectional contextual representations, allowing fine-tuning for specific tasks (Devlin *et al.* 2018). This success expanded into genomics with DNABERT, a BERT-based model pre-trained on human genome sequences, enabling contextual embeddings for tasks such as promoter detection (e.g. DNABERT-Prom) (Ji *et al.* 2021). Nucleotide Transformer was later developed to effectively capture sequence dependencies (Dalla-Torre *et al.* 2024), followed by DNABERT-2, a model with enhanced tokenization and attention mechanisms, reducing resource expenditure (Zhou *et al.* 2023). Transformer-based models have not been evaluated in bacterial promoter predictors for multiple species.

Regardless of the great advances in promoter identification using ML-based predictors, negative dataset selection and its impact on classification have been scarcely evaluated. Promotech used negative sequences extracted from genomic regions lacking previously reported promoters, while TIMER and iPro-WAEL extracted random genomic sequences followed by a filtering step (Chevez-Guardado and Peña-Castillo 2021, Zhang *et al.* 2022, Zhu *et al.* 2023). Single-species predictors (e.g. iPro70-FMWin, 70ProPred, CNNProm) generally use random sequences from coding, non-coding, or intergenic regions, but no consensus has been established. Cassiano and Silva-Rocha (2020) systematically compared promoter prediction tools for *E. coli*, evaluating detection metrics against a pool of true promoters and negative sequences generated randomly with an AT distribution similar to promoters. Benchmarked tools achieved regular detection performance, as reflected in accuracy values between 0.72 and 0.76 and specificity (Sp) from 0.51 to 0.69, suggesting a propensity to detect false positives (FP). The authors attribute this outcome to a bias involving low AT (higher GC-content) in negative sequences used as training datasets, evidenced by high variability in Sp values. GC-content bias in negative datasets has only been suggested in *E. coli* by Cassiano and Silva-Rocha (2020), while multiple-species models count with a larger GC-content diversity which can reinforce this bias. Besides, data from experimentally validated promoter sequences from different species are scarce, with large differences from tens to thousands between species datasets (Su *et al.* 2021).

To address the aforementioned challenges, the aims of this study are: (i) to evaluate whether GC-content classification bias exists in multiple-species ML-based models using a negative dataset extracted from CDS; (ii) to assess the feasibility of reducing bias by generating synthetic random sequences (SRS) that simulate the GC-content distribution of the promoters available for each species; (iii) to evaluate the effectiveness of LLMs in the context of promoter classification across various species through a single model.

## 2 Materials and methods

Designing ML classifiers for promoter detection presents a challenge due to the difficulties in creating a balanced dataset. While obtaining bacterial promoter sequences (positive dataset) from curated databases, such as RegulonDB (Salgado *et al.* 2023) or the Prokaryotic Promoter Database (PPD) (Su *et al.* 2021), is straightforward and has been used previously (Chevez-Guardado and Peña-Castillo 2021), formulating an appropriate set of non-promoter (negative dataset) sequences for training is still not standard. Here, we inquire into the effectiveness of two strategies for generating negative datasets: extracting non-promoter sequences from CDS of each strain genome and synthesizing random sequences (SRS) artificially to counterbalance GC-content distribution of the positive dataset. Although synthetic data could cause model overfitting, previous works in other subjects have used this approach to generate balanced datasets with good results in real data (Gao *et al.* 2023).

**Figure 1.**
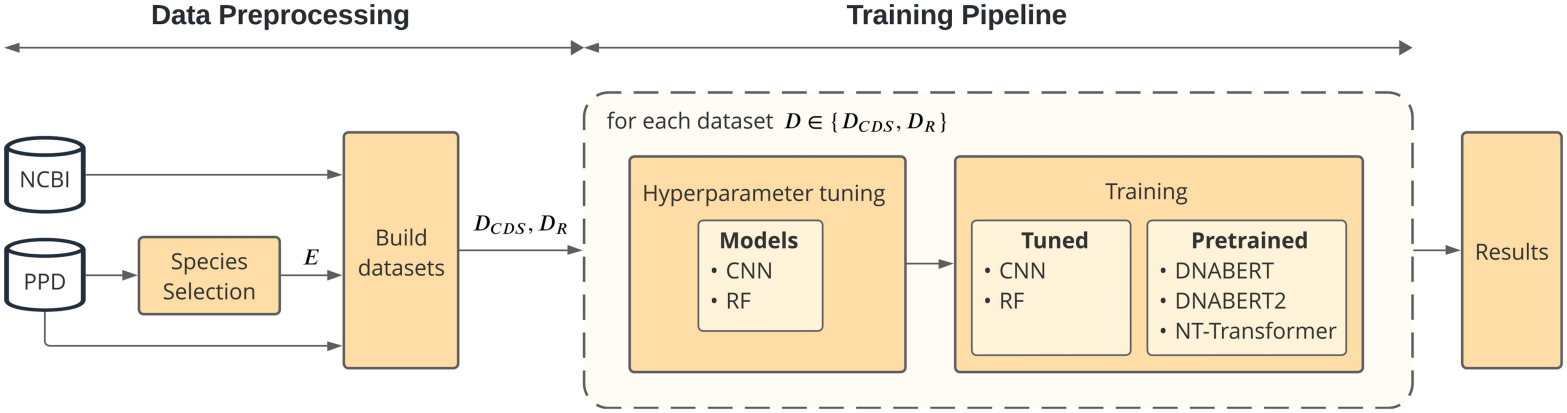
General overview of the framework for this study. Data pre-processing stage includes species selection, promoters extraction from PPD, and CDS retrieved from NCBI to build negative datasets. Training pipeline comprises tuning hyperparameters for CNN and RF, and training stages for pretrained (DNABERT, DNABERT-2, Nucleotide Transformer) and tuned models (CNN, RF).

The general framework comprises a data pre-processing stage, followed by a training stage (Fig. 1). Species included were selected based on the promoters available in the PPD according to criteria such as the number of sequences, evolutionary relatedness, and GC-content. Two datasets were developed for general assessment of ML models, both sharing the promoter data differing in the construction of the non-promoter sequences. Non-promoter sequences were randomly extracted from coding sequences (CDS) or composed of SRS based on species-specific GC-distribution of the promoter data. Following this, a training workflow was applied for each model.

### 2.1 Species selection

PPD includes promoter data of bacterial and archaeal species, comprising many entries with <200 promoter sequences per organism. Only species with >200 promoters were pre-selected for this study (Supplementary Table S1). To ensure evolutionary relatedness between promoter sequences, we only selected species from one phylum, *Pseudomonadota*, which exhibited the largest number of promoter sequences (*n* = 80 448), and a greater diversity in GC-content and genera to enrich the robustness and sequence representation within the positive dataset.

### 2.2 Dataset pre-processing

**Figure 2.**
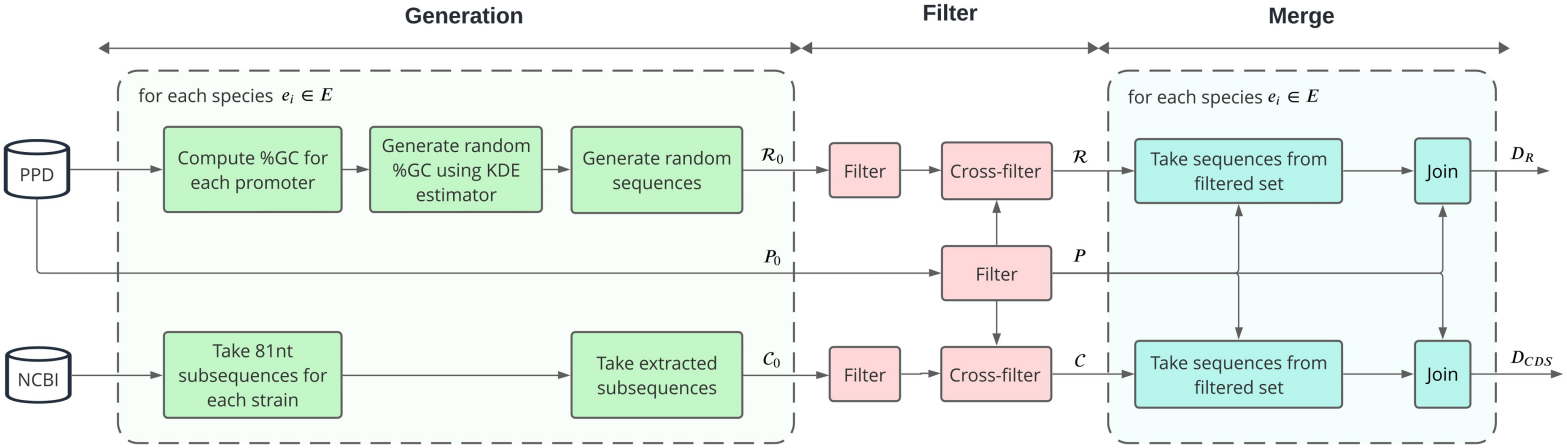
Dataset pre-processing workflow. Promoters sequences were extracted from PPD. Non-promoter sequences were extracted from CDS (NCBI), or synthetically generated (SRS), followed by a filtration step removing redundant sequences. A final merging stage is performed for each species.

Promoters (81-nt, -60 and +20 nt from the transcription start site, TSS) were obtained from PPD, including all the sequences from each *Pseudomonadota* strain with >200 promoters ($P_{0}$; Fig. 2). For the CDS negative dataset, random 81-nt were extracted from the CDS of each genome assembly (NCBI) associated with the strains included in the positive dataset (Su *et al.* 2021). For the SRS negative dataset, the Kernel Density Estimator technique was used to approximate a probability distribution function of GC-content based on the promoters available in $P_{0}$ per species included ($e_{i}$); then, the same function produced GC-content values delivered to a random generator algorithm to create the SRS dataset (length = 81-nt; Supplementary Algorithm S1). In both negative datasets (CDS and SRS), twice as many sequences were extracted for each species in comparison to the positive dataset. This ensures an equal or greater number of sequences in the negative dataset available after filtering, allowing for balanced promoter and non-promoter sequences in subsequent phases without compromising promoter data. Following this phase, the raw non-promoter sequence sets $C_{0}$ and $R_{0}$ were obtained for CDS and SRS, respectively.

Initially, a filtering operation is applied within each raw dataset ($P_{0}$, $C_{0}$, and $R_{0}$) to avoid repeated sequences. Subsequently, a cross-filtering operation is performed between the promoter and non-promoter datasets. This step filters out non-promoter sequences similar to the promoters P, resulting in filtered non-promoter C and R sets (Supplementary Table S2). The entire process utilizes the CD-HIT program (Fu *et al.* 2012) with a threshold of 0.8 to ensure effective redundancy reduction (Le *et al.* 2022, Zhu *et al.* 2023). Following the filtering stage, promoter and non-promoter data were integrated based on their respective origins. For each species strain, non-promoter sequences were extracted from C and R. The quantity of non-promoter sequences extracted matches the number of available filtered promoters after the filtering phase, ensuring a balanced dataset between positive and negative datasets. Subsequently, the non-promoter sequences were combined with the extracted sequences to form the datasets $D_{\mathrm{CDS}}$ and $D_{R}$.

### 2.3 Training pipeline

*Model selection.* We evaluated Random Forest (RF), Convolutional Neural Networks (CNN), and BERT-based architectures for promoter prediction. RF models aggregate multiple decision trees to form an ensemble, enhancing robustness and reducing overfitting. CNN, optimized for grid-like data such as DNA sequences, were also used to identify promoter regions. BERT-based models, including DNABERT, DNABERT-2, and Nucleotide Transformer, were used to effectively interpret nucleotide sequences, addressing the unique challenges of genomic data analysis. Detailed configurations for each model are provided in Supplementary Note S1.

*Input encoding.* For both the CNN and RF models, the sequences in the dataset were encoded using one-hot encoding. In this scheme, each nucleotide was distinctly mapped to a 4D vector: A→(1,0,0,0), C→(0,1,0,0), G→(0,0,1,0), and T→(0,0,0,1). For a sequence of length N, the CNN uses a (N,4) encoding, while the RF model utilizes a flattened 1D vector of length 4N. For BERT-based models, they integrate positional encoding and embeddings within the attention mechanism, enabling them to discern context and dependencies across extended segments of the sequence.

*Hyperparameter tuning.* RF, CNN, and BERT-based architectures adapted for DNA sequences were utilized, with hyperparameter tuning applied exclusively to RF and CNN models. Conversely, as LLMs are pre-trained on extensive datasets, BERT-based models exhibit robust performance without the need for substantial hyperparameter adjustments. The dataset is initially divided into 80% for training and 20% for validation. During hyperparameter tuning, the training set (80% of the data) is further divided into k-folds, with each fold split into 80% for training and 20% for validation within the tuning process. Detailed hyperparameter tuning procedures and model configurations for RF and CNN are provided in Supplementary Note S2.

*Training.* In the training stage, RF and CNN models were trained using the best hyperparameters identified during the hyperparameter tuning. The validation set, reserved from the initial 80/20 split of the dataset, was used for evaluating all models, including the RF, CNN, and BERT-based architectures. Detailed training aspects are provided in Supplementary Note S3.

*Performance evaluation.* For classification model evaluation, key metrics were used to measure performance. Metrics evaluated include specificity (Sp), sensitivity (Sn), precision (Pre), accuracy (Acc), Matthews correlation coefficient (MCC), F1 score ($F_{1}$), and the area under the ROC curve (ROC AUC).

## 3 Results

### 3.1 Bacterial species selection for multiple-species models

Due to the high diversity within bacteria, to improve the promoter prediction performance, we selected a single phylum to focus on in this study. Previous studies have not taken into account taxonomic information to select evolutionary relatedness between species. *Pseudomonadota* harbored the greatest amount of data (80 448 promoter sequences) and species (10), with each species belonging to a different genus, improving the diversity of the dataset in comparison to other phyla (Supplementary Table S1).

### 3.2 Characterization of promoter and non-promoter datasets based on GC-content

**Figure 3.**
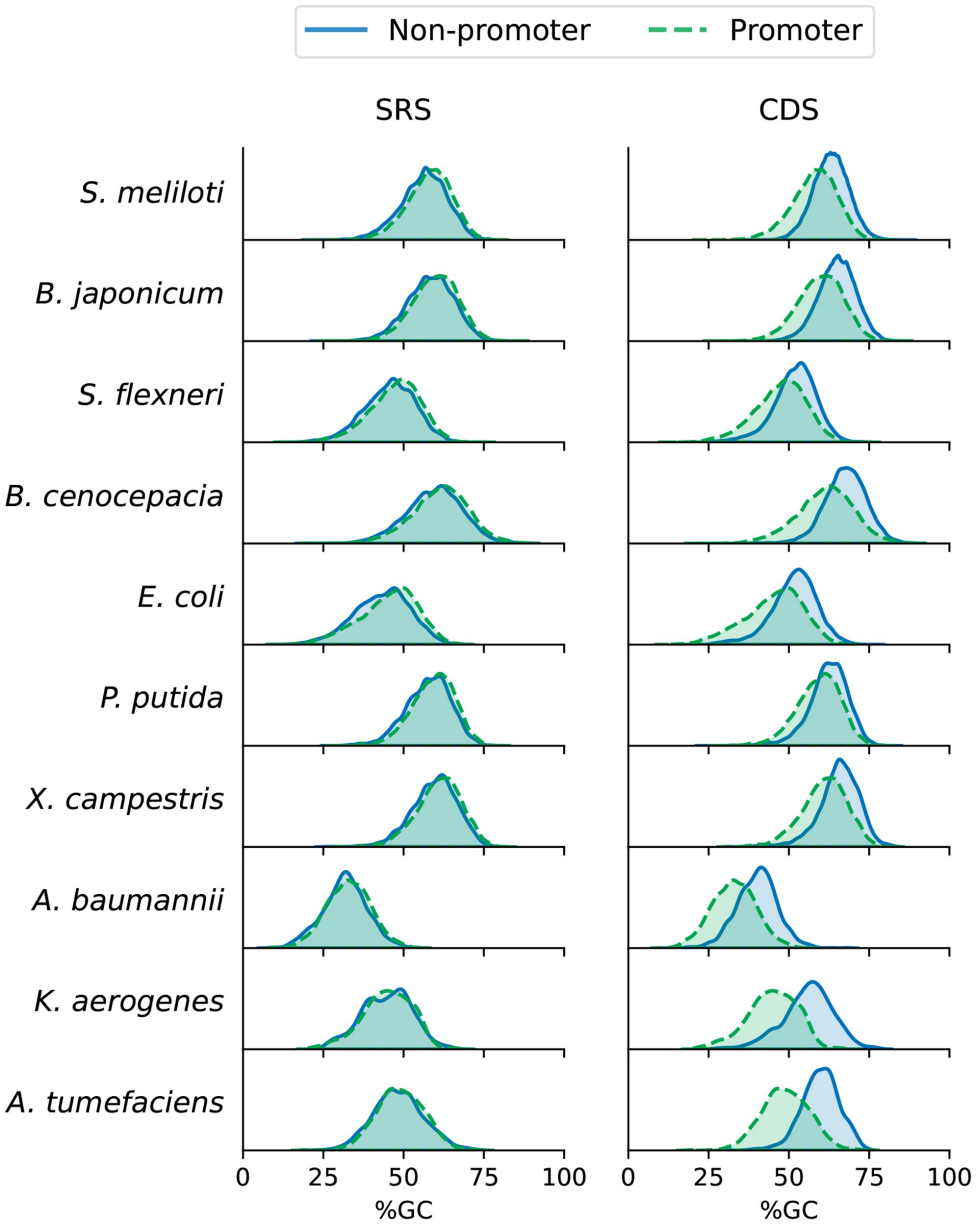
GC-content distribution of promoter and non-promoter sequences in the dataset.

After filtering redundant sequences, 65 647 promoters were available for dataset construction (Supplementary Table S2). Regarding non-promoters (SRS and CDS), in all the species included, the number of sequences exceeded the positive dataset, enabling the creation of balanced datasets per species. Mean GC-content and GC-content distribution of the positive and negative datasets were analyzed per species according to their origin (Fig. 3 and Supplementary Table S3). GC-content distributions of promoter sequences showed differences between species, observing the lowest distribution for *Acinetobacter baumannii* (mean GC-content: 32.90%), and the highest for *Burkholderia cenocepacia* (mean GC-content: 61.41%). Overall, promoter sequences showed a negative skew in GC-content distribution, indicating the majority of the dataset is composed of a higher GC-content (mean GC-content: 56.05%). In the case of the CDS negative dataset, GC-content distribution between the positive and negative datasets varies in a species-dependent manner. For example, in the species *Klebsiella aerogenes* and *Agrobacterium tumefaciens*, the distributions differ greatly (Fig. 3; ΔGC-content: 11.04% and 10.77%, respectively), whereas *Pseudomonas putida* showed closer similarity (Fig. 3; ΔGC-content: 3.03%). Overall, CDS non-promoter sequences showed a more pronounced negative skew in GC-content distribution than promoters, with a 5.19% higher mean GC-content than the positive dataset (Supplementary Table S3). Conversely, in the SRS negative dataset, promoter and non-promoter sequences share a similar GC-content distribution and mean in all the species (Fig. 3; ΔGC-content < 2.0%). This behavior was also observed in the validation subset used for model performance (Supplementary Fig. S1).

### 3.3 Comparative performance evaluation of models trained on SRS or CDS datasets

A performance comparison of the models for each negative dataset studied (CDS and SRS) was evaluated in terms of specificity (Sp), sensitivity (Sn), recall (Pre), accuracy (Acc), MCC, F1 Score, and overall ROC AUC (Table 1). DNABERT and Nucleotide Transformer exhibited superior performance in both negative datasets, demonstrating a higher learning capacity in both scenarios. Within BERT-based models, DNABERT-2 is outperformed by DNABERT and Nucleotide Transformer.

**Table 1.** Overall performance metrics for ML-based models for promoter predictors in multiple-species using CDS and SRS as negative datasets. Bold values indicate the best-performing model for each dataset.

Dataset	Model	Sp	Sn	Pre	Acc	MCC	$F_{1}$	ROC AUC
CDS	RF	0.7761	0.6440	0.7448	0.7096	0.4237	0.6907	0.7824
	CNN	0.8574	0.7307	0.8387	0.7936	0.5925	0.7810	0.8678
	DNABERT	0.8665	**0.7660**	0.8534	**0.8159**	**0.6355**	**0.8074**	**0.8894**
	DNABERT-2	0.8763	0.6851	0.849	0.78	0.5715	0.7583	0.8517
	Nucleotide Transformer	**0.8885**	0.7402	**0.8707**	0.8138	0.6353	0.8002	0.89
SRS	RF	0.8099	0.9520	0.8356	0.8815	0.7704	0.89	0.9554
	CNN	0.9374	0.9325	0.9379	0.9349	0.8698	0.9352	0.9853
	DNABERT	0.9264	0.9666	0.9302	**0.9466**	**0.894**	**0.948**	**0.9885**
	DNABERT-2	0.9125	**0.9691**	0.9182	0.941	0.8833	0.943	0.9867
	Nucleotide Transformer	**0.9439**	0.9462	**0.9448**	0.945	0.8901	0.9455	0.9872

For models trained with the CDS dataset, DNABERT performed best across most metrics. All CDS-trained models exhibit relatively high Sp compared to Sn, indicating a gap that favors the accurate classification of non-promoters. The imbalance seen in every model may suggest that the issue is mainly associated with the input data, pointing to potential improvements in the dataset construction as a crucial step. For ML-based models trained with the SRS dataset, the values across all metrics significantly increase by 15–20 points compared to the CDS dataset, with DNABERT and Nucleotide Transformer outperforming all other models. The difference between the two models in Sp, Sn, and recall remains consistent at 1–2 points, highlighting their performance across different datasets. Regarding Sn and Sp, the predictors exhibit a more balanced performance, with a less pronounced gap between these metrics than models trained with the CDS dataset. ML-models using SRS as a negative dataset identify promoters and non-promoters with similar performance (Table 1).

### 3.4 GC-content of the negative dataset affects specificity–sensibility in a species-dependent manner

ML models trained with the CDS dataset have a detection preference for non-promoter than promoter sequences. Due to the multiple-species character of these models, we evaluated which species could be more susceptible by the learned preference in the overall best model. From the five models tested, the DNABERT predictor was chosen the best model across four out of seven metrics (Table 1). Even though the overall metrics for DNABERT-based model using CDS showed a higher Sp (0.8665) than Sn (0.7660), that pattern is not observed in every species (Supplementary Table S4). For example, *A. baumannii* showed the highest Sn (0.9685) and lowest Sp (0.7512), suggesting that the model is preferably classifying promoter than non-promoter sequences in *A. baumannii*. Interestingly, *A. baumannii* have the lowest GC-content for promoter and non-promoter sequences of the dataset, suggesting that sequences rich in AT are easy to classify as promoters and hard to identify as non-promoter than the rest of the dataset (Fig. 3). This behavior also can be observed for *K. aerogenes*, with a low GC-content in the promoter dataset. Coinciding with this finding, sequences of other species rich in GC, such as *B. cenocepacia* and *Xanthomonas campestris*, showed a high Sp (>0.914), a better classification of non-promoters. Other species present subtle differences of about 0.01 up to more significant disparities of around 0.22, further emphasizing the heterogeneity in detection capabilities across different organisms in multiple-species predictors. Moreover, species such as *E. coli*, *Sinorhizobium meliloti*, and *P. putida* notably diminish the dataset Sn, each showing values <0.685, and *Bradyrhizobium japonicum* with a Sn of 0.774. For the latter species, only *P. putida*, *S. meliloti*, and *B. japonicum* possess GC-rich sequences in the positive training data, whereas *E. coli* does not follow a trend explained by GC-content. These results illustrate that GC-content could be in part responsible for bias in ML promoter classifiers for multiple-species, but other traits (e.g. tetranucleotide frequency or other sequence features) could also contribute to this bias.

### 3.5 Reduced sensitivity in the BERT-model trained with CDS dataset

**Figure 4.**
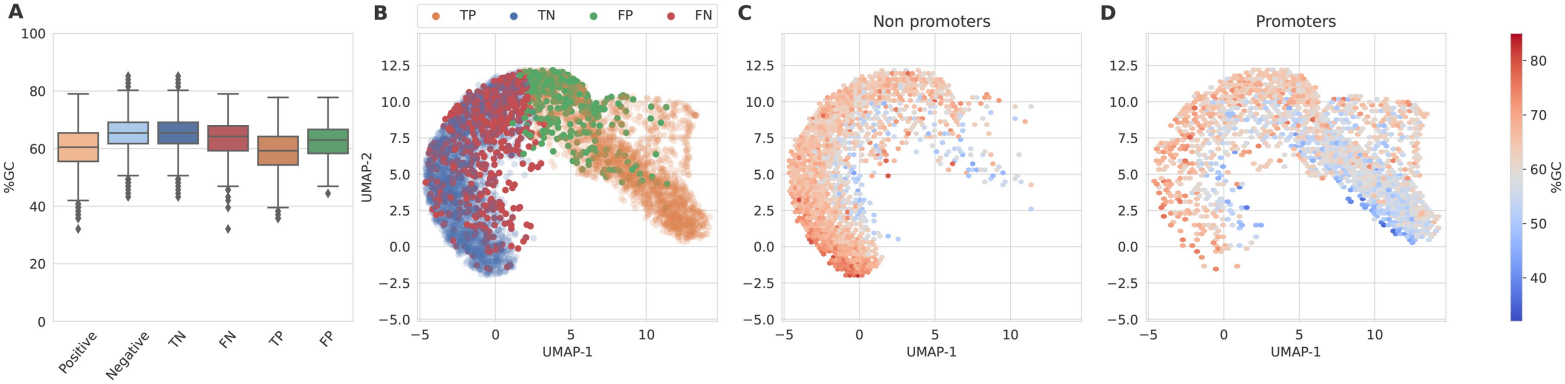
Comparative analysis for *B. japonicum* classifications in the DNABERT-based model trained with the CDS dataset. (A) Boxplots of GC-content distribution for positive and negative datasets along with predicted categories (TP, TN, FP, FN). (B) UMAP projections of the [CLS] token from DNABERT, based on validation data. (C, D) UMAP projections for non-promoters (TN+FP) and promoters (TP+FN) ground truth, respectively, each augmented by density heatmaps of GC-content.

As Cassiano and Silva-Rocha (2020) highlighted in *E. coli* predictors, some tools for promoter detection are bias toward rich AT-sequences. To further analyze the observations based on Sp and Sn and their relationship to GC-content of the DNABERT-based model trained with CDS, a latent space projection of the [CLS] output tokens was performed using Uniform Manifold Approximation and Projection (UMAP) (McInnes *et al.* 2018). UMAP reduces the dimensionality of the data, facilitating the visualization and analysis of complex high-dimensional data as part of the validation analysis. UMAP projections of promoter and non-promoter sequences (Fig. 4C and D) showed that the classification task possesses GC-content variations between the prediction clusters. The non-promoter cluster (TN + FP; Fig. 4C), ranging predominantly from 60% to 80% GC-content, is more distinctly defined compared to the promoter cluster (TP + FN; Fig. 4D), which shows greater dispersion within the model projection and spans mainly from 40% to 60% GC-content. This overlap is visible in Fig. 4B, where many FN overlap with TN, highlighting challenges in category differentiation. Notably, many promoters, especially those with higher GC-content levels, are found within the non-promoter region of the model projection. Conversely, fewer non-promoters appear within the promoter cluster, indicating classification asymmetry and dispersion that suggest difficulty distinguishing promoter sequences. The prominence of higher GC-content among misclassified promoters within the non-promoter cluster further evidences a bias, impacting classification accuracy. These findings are also found in other species such as *B. cenocepacia*, *E. coli*, *Shigella flexneri*, *S. meliloti*, and *P. putida* (Supplementary Fig. S2). GC-content distribution across actual datasets and predictions presents a notable overlap between FN and TN with both categories showing considerable similarity in their interquartile ranges (Fig. 4A). TN have a GC-content range from ∼62% to 69%, centering around a median of 65%. FN shows a slightly narrower interquartile range from about 59% to 68%, with a median of 64%. This proximity in distribution suggests that the DNABERT-based model often misclassifies genuine promoter sequences as non-promoters when their GC-content falls within these overlapping ranges, suggesting a bias in the model ability to differentiate based on GC-content. Interestingly, FN tends to overlap with TN in other species as well (Supplementary Fig. S2). Distributions for TP and FP present less overlap and possess a distinct range, highlighting differences in the model sensitivity to other promoter intrinsic characteristics (Supplementary Fig. S2).

### 3.6 Synthetic non-promoter sequences improve bias reduction in promoter predictors

**Figure 5.**
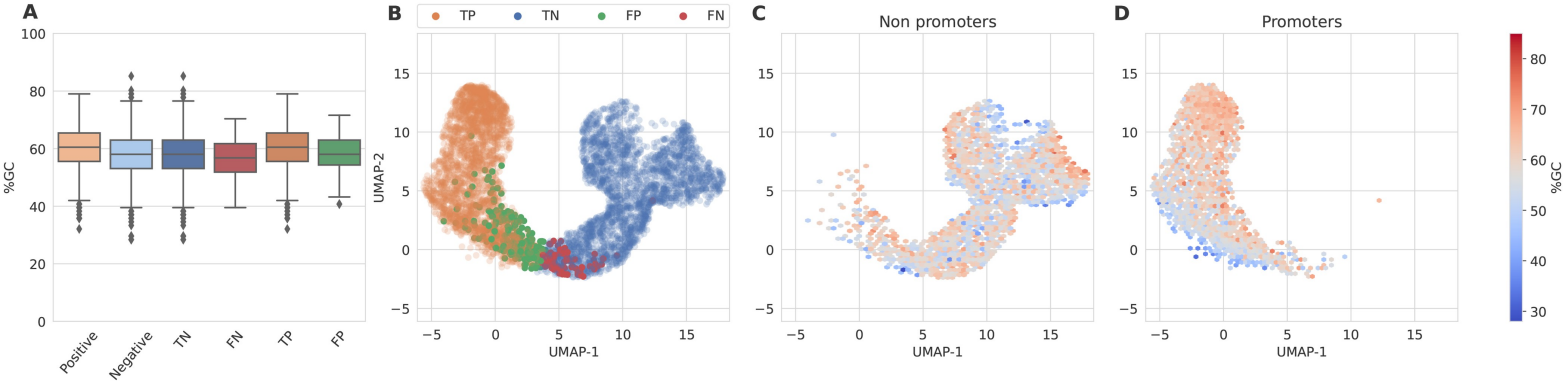
Comparative analysis for *B. japonicum* classifications in the DNABERT-based model trained with the SRS dataset. (A) Boxplots of GC-content distribution for positive and negative datasets along with predicted categories (TP, TN, FP, FN). (B) UMAP projections of the [CLS] token from DNABERT, based on validation data. (C, D) UMAP projections for non-promoters (TN+FP) and promoters (TP+FN) ground truth, respectively, each augmented by density heatmaps of GC-content.

A notable improvement is observed for the SRS-trained model using DNABERT compared to the CDS-trained model; Sp and Sn gaps are smaller, with no more than a 0.05-point difference per species (Supplementary Table S5). This suggests that SRS effectively leads to more balanced models in terms of promoter and non-promoter predictions. Furthermore, Sn generally exceeds Sp, indicating enhanced promoter identification. Dimensionality reduction enabled a detailed visualization of how SRS influences the model classification ability (Fig. 5). GC-content distribution across classifications showed less variability in GC-content ranges than the CDS dataset (Fig. 5A). TN and FN display overlapping interquartile ranges from ∼53% to 62%, suggesting a narrower distribution gap. Similarly, TP and FP have interquartile ranges from 56% to 63%, indicating overlapping distributions without a clear bias in GC-content for any group. This pattern suggests a more uniform handling of GC-content across categories than the CDS dataset. Figure 5B showcases UMAP projections for the SRS dataset classified by predicted categories, whereas Fig. 5C and D represent the non-promoter and promoter clusters, respectively. While some FN and FP overlap within their respective clusters, there is no discernible pattern of GC-content significantly distinguishing the clusters. The absence of a clear GC-content pattern between the promoter and non-promoter prediction clusters suggests a reduction in the GC-content-related bias previously noted in the CDS dataset. These findings are consistent across other species included (Supplementary Fig. S3). Although the SRS dataset contributes to more balanced and accurate classification outcomes in the validation data, this result should be taken carefully as a result of model overfitting. To test this, we evaluated the behavior of the CDS- and SRS-trained models in real genomic data of the species with the best metrics, *X. campestris*. Genomic locus of ten promoters from *X. campestris*, including 5000 nt upstream and downstream from the TSS, were analyzed by a sliding window with a stride of 1 nt to predict promoter and non-promoter regions depicted by average softmax probability (Supplementary Figs S4 and S5). We considered probability variation as a promoter detection signal. The CDS-trained model showed inconsistent behavior with sudden probability variations throughout the entire sequence, detecting most promoters with high false positives. The SRS-trained model exhibited a consistently high detection signal across the genomic sequence (P=1). However, the latter displayed some negative perturbations close to promoter regions suggesting an inverted classification model (1-P).

## 4 Discussion

For the proper development and improvement of ML-based tools in sequence analysis, more insight about the training datasets that we use to create predictive models must be addressed. Due to our narrow knowledge of promoter sequences in multiple bacterial species, with only a few strains with enough data, it is challenging to be aware of which factors may create bias. Bias is not uncommon in ML-based tools, with several stages being able to cause unfairness in the task pursued. This includes: dataset selection, hyperparameters optimization, algorithms, or model architecture chosen (Bischl *et al.* 2023). Here, we focus on dataset curation, particularly data-driven biases caused by the negative dataset.

GC-content variation is intrinsic to any genomic sequence, which highlights the hindrance to create a proper negative dataset for any ML-based tool for single or multiple species. Bacterial GC-content ranges from 16% to 77%, with 90% of organisms between 33% and 71% (Aliperti *et al.* 2023). GC-content standardization for negative datasets has been taken into consideration in a few models, e.g. in the assessment of methods to identify cis-regulatory motifs (Zhang *et al.* 2021). Nonetheless, correction of negative datasets by GC-content has not been investigated in predictors outcomes. Multiple-species predictors are inherently variable in model performance across species depending on the negative dataset chosen, with GC-content as one factor to consider. For CDS-trained models, the observed gaps in Sp and Sn (>20 points) highlighted the need for further refinement. Performance evaluation in independent datasets of multiple-species predictors (TIMER, Promotech, iPro-WAEL, iPro70-FMWin) also showed this problem, with large Sn and Sp gaps per species evaluated (Zhu *et al.* 2023). Here, the SRS dataset outperformed the CDS-trained models, in particular improving the gaps between Sn and Sp. Even though the usage of synthetic datasets could cause model overfitting, the applicability and reliability of these models in real data need to be tested to ensure the generation of models with real-world applications. In this study, both models showed average behavior in real genomic data, stressing the need to validate these models in genomic sequences.

In recent years, many BERT-based tools have improved predictor performance in sequence analysis compared to other ML or deep-learning techniques in Eukarya and Bacteria. DNABERT-Prom, BERT-Promoter, msBERT-Promoter are examples of these models using the Attention mechanism, generally outperforming previous models based on CNN or less complex architectures (Ji *et al.* 2021, Le *et al.* 2022, Li *et al.* 2024). In this study, DNABERT was the overall best model in both datasets. For SRS, DNABERT remained the best model; however, the GC-content normalization enabled less complex architectures such as CNN and RF models to become competitive in performance metrics with BERT-based models despite their lower computational complexity. Although large computational resources are required for NLP models like BERT-based models, this underscores the relevance of CNN and RF models for multi-species promoter prediction in GC-content matched sequences.

## Supplementary Material

btaf135_Supplementary_Data

## Data Availability

The source code and datasets of the experiments performed in this study are freely available at https://github.com/maigonzalezh/MultispeciesPromoterClassifier, and have been archived on Zenodo with DOI 10.5281/zenodo.15016403.
