# Supplementary material for: Negative dataset selection impacts machine learning-based predictors for multiple bacterial species promoters
Source: Bioinformatics. 2025 Mar 27;41(4):btaf135. doi: 10.1093/bioinformatics/btaf135 (PMC11993300; doi:10.1093/bioinformatics/btaf135)
Supplement: btaf135_Supplementary_Data [file btaf135_supplementary_data.pdf]

Supplementary Data

Supplementary Note S1: Model Selection

In this study, we employed various machine learning and deep learning methodologies for sequence analysis. Below, we briefly describe each model:

Random Forest (RF)

RF models are supervised learning algorithms that integrate multiple decision trees to form an ensemble. Each tree is built from a random subset of data and variables, which enhances robustness and reduces the risk of overfitting. This method is widely used for classification and regression tasks, providing detailed feature importance metrics that aid in model interpretation.

Convolutional Neural Network (CNN)

CNNs are deep learning algorithms optimized for analyzing grid-like data structures, such as DNA sequences, to identify promoter regions. The CNN architecture used in this study includes three one-dimensional convolutional layers designed for linear sequence data, each followed by batch normalization to stabilize training and max pooling to reduce spatial dimensions and enhance feature detection. Dropout layers are incorporated to prevent overfitting, and a flattened layer converts multi-dimensional outputs into a one-dimensional array. Finally, the network includes three dense layers with ReLU activation and a single dense output layer with a sigmoid activation function to predict promoter regions' presence accurately.

BERT-based Models

In BERT-based models, the `[CLS]` token is placed at the start of an input sequence to collect and summarize contextual information from the entire sequence using the model's attention mechanism. This information is then processed through a dense layer with ReLU activation, followed by a sigmoid output layer that generates a probability score for classification tasks. For the experiments conducted in this study, specific versions of DNABERT, DNABERT-2, and Nucleotide Transformers were selected:

- DNABERT-3, which features approximately 100M parameters and employs 3-mer tokenization.
- Nucleotide Transformer v2, a multi-species version with 500M parameters.
- DNABERT-2, utilizing the only available model with 117M parameters.

Supplementary Note S2: Hyperparameter tuning

Convolutional Neural Network (CNN)

The hyperparameters are optimized using the Optuna algorithm over 60 trials included the dropout rate, which was varied from 0.2 to 0.8 in increments of 0.1, the number of neurons in each convolutional layer, which was set to 128, 256, or 512, and the kernel sizes, which were chosen from 3, 5, or 7. Additionally, L2 regularizers were applied to the weights and biases in each convolutional layer, with possible values of 0.001 or 0.0001. For the dense layers, the number of neurons was set to 128, 256, or 512, and L2 regularization was similarly tuned with values of 0.001 or 0.0001.

Random Forest (RF)

The Random Forest model was tuned using a grid search algorithm, aiming to maximize the mean F1 score. The hyperparameters explored included the number of features considered for each split, which was set to either "sqrt" or "log2", and the number of estimators, which varied between 1000, 2000, 3000, 4000, and 5000.

Supplementary Note S3: Training

For CNN models, an Adam optimizer with a learning rate of 1e-4 was used, while BERT-based models employed an AdamW optimizer with a weight decay of 0.01. A range of learning rates $[1e^{-5}, 2e^{-5}, 3e^{-5}, 4e^{-5}, 5e^{-5}]$ was tested, with the optimal rate selected based on the highest F1 score on the validation data.

Supplementary Algorithm S1 Generation of a Random Sequence with Fixed GC Content

Require: *L*: Desired length of the sequence
Require: *GC%*: Desired percentage of G and C nucleotides in the sequence
Ensure: *sequence*: A random sequence of length *L* with an approximate GC percentage

Round *GC%* to the nearest integer and assign to *gc_rounded*
$at\_rounded \leftarrow 100 - gc\_rounded$
if $gc\_rounded = 0$ **then**
    $g\_percentage \leftarrow 0$
else
    $g\_percentage \leftarrow$ random integer between 0 and *gc_rounded*
end if
$c\_percentage \leftarrow gc\_rounded - g\_percentage$
if $at\_rounded = 0$ **then**
    $a\_percentage \leftarrow 0$
else
    $a\_percentage \leftarrow$ random integer between 0 and *at_rounded*
end if
$g\_count \leftarrow \left\lfloor L \cdot \frac{g\_percentage}{100} \right\rfloor$
$c\_count \leftarrow \left\lfloor L \cdot \frac{c\_percentage}{100} \right\rfloor$
$a\_count \leftarrow \left\lfloor L \cdot \frac{a\_percentage}{100} \right\rfloor$
$t\_count \leftarrow L - g\_count - c\_count - a\_count$
Construct the initial string *sequence* as the concatenation of the following substrings:
    (i) A substring of *g_count* characters, each being ’g’.
    (ii) A substring of *c_count* characters, each being ’c’.
    (iii) A substring of *a_count* characters, each being ’a’.
    (iv) A substring of *t_count* characters, each being ’t’.
Randomly shuffle the characters in *sequence* to obtain the final sequence.
return *sequence*

Supplementary Table S1. Number of promoters per phylum and species.

Phylum	Specie	N° promoters
Pseudomonadota	*Acinetobacter baumannii* ATCC 17978	1540
	Agrobacterium tumefaciens str C58	706
	Bradyrhizobium japonicum USDA 110	15933
	Burkholderia cenocepacia J2315	10831
	Escherichia coli str K-12 substr. MG1655	8616
	Klebsiella aerogenes KCTC 2190	763
	Pseudomonas putida strain KT2440	7938
	Shigella flexneri 5a str. M90T	14051
	Sinorhizobium meliloti 1021	17003
	Xanthomonas campestris pv. *campestris* B100	3067
	**Total**	**80448**
Cyanobacteria	*Nostoc* sp. PCC7120	13705
	Synechococcus elongatus PCC 7942	1473
	Synechocystis sp. PCC 6803	944
	**Total**	**16122**
Campylobacterota	*Campylobacter jejuni* RM1221	2166
	Campylobacter jejuni subsp. *jejuni* 81-176	2142
	Campylobacter jejuni subsp. *jejuni* 81116	1942
	Campylobacter jejuni subsp. *jejuni* NCTC 11168	1905
	Helicobacter pylori strain 26695	2228
	**Total**	**10370**
Bacillota	*Bacillus subtilis* subsp. *subtilis* str. 168	691
	Paenibacillus riograndensis SBR5	2351
	Staphylococcus aureus subsp. *aureus* MW2	2821
	Staphylococcus epidermidis ATCC 12228	2207
	Streptococcus pyogenes strain S119	892
	**Total**	**8962**
Euryarchaeota	*Haloferax volcanii* DS2	4749
	Thermococcus kodakarensis KOD1	2720
	**Total**	**7469**
Actinomycetota	*Corynebacterium diphtheriae* NCTC 13129	1656
	Corynebacterium glutamicum ATCC 13032	3581
	**Total**	**5237**
Mycoplasmatota	Onion yellows phytoplasma OY-M	231
	**Total**	**231**

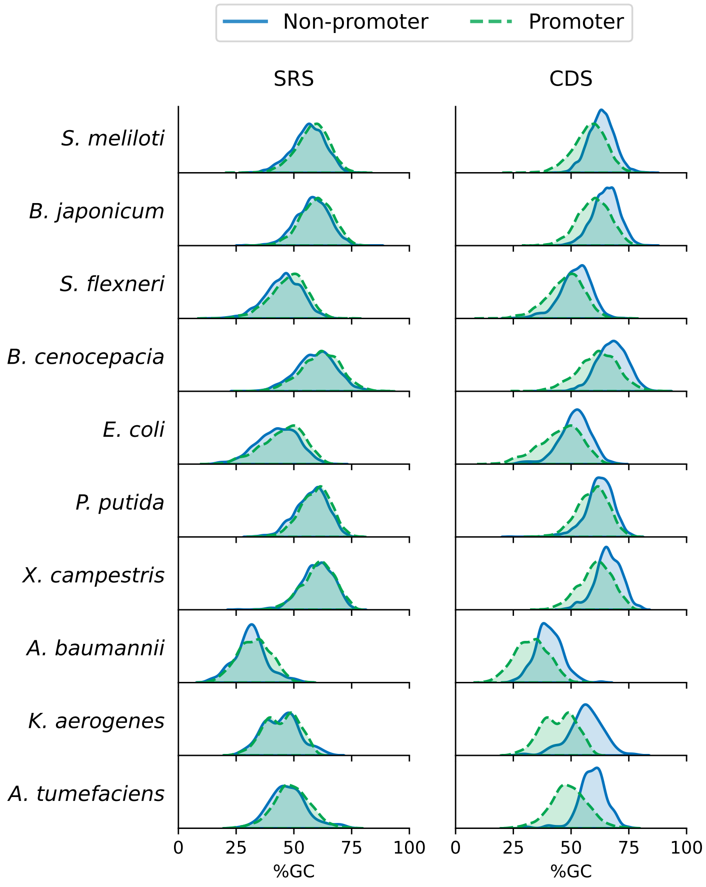

Supplementary Figure S1: GC-content distribution of promoter and non-promoter sequences in the validation dataset.

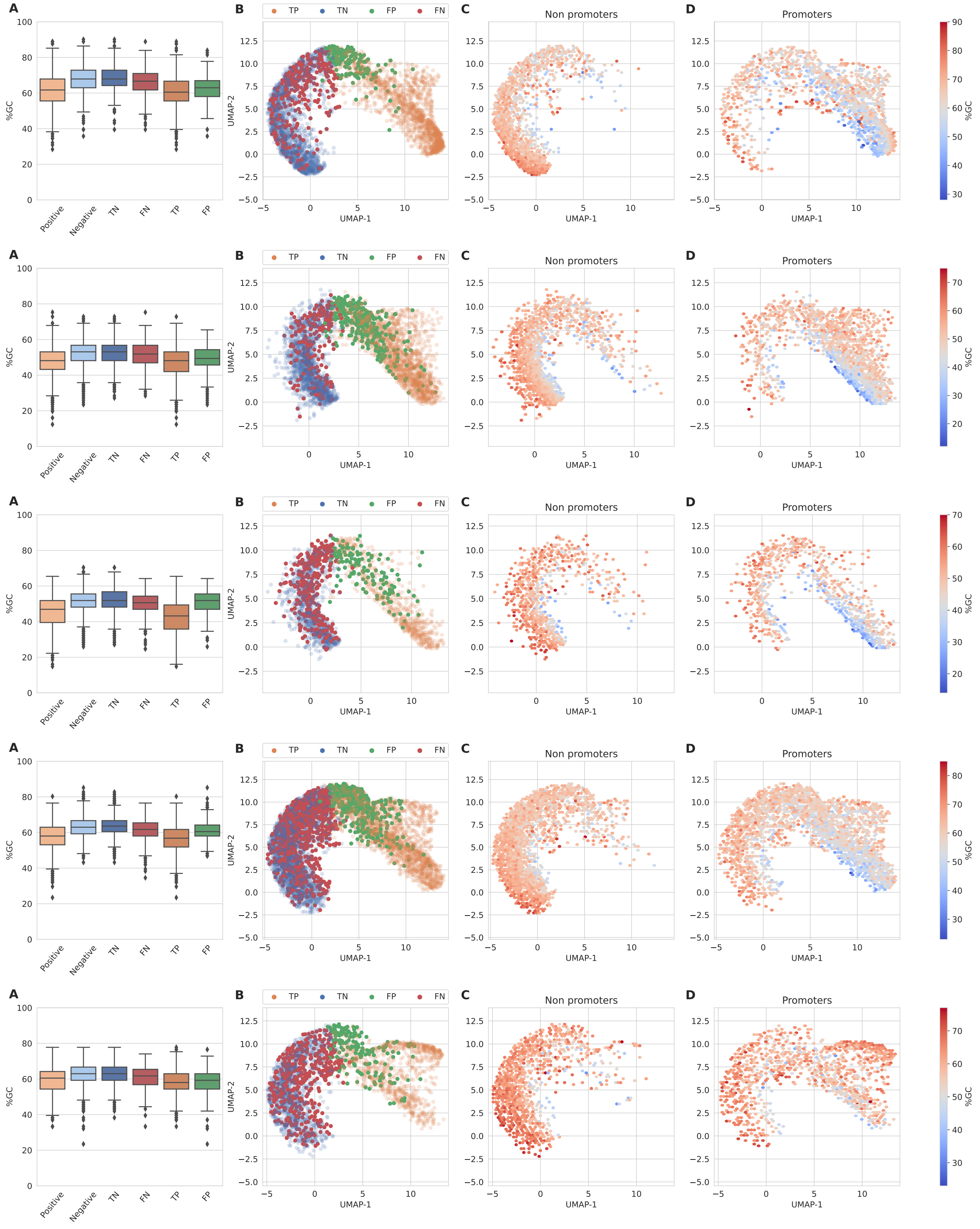

Supplementary Figure S2: **Comparative Analysis of Genomic Sequence Classifications in CDS dataset**. **A**: Boxplots of GC content distribution for actual positive and negative classifications along with predicted categories (TP, TN, FP, FN). **B**: UMAP projections of the `[CLS]` token from DNABERT, based on validation data. **B,D**: feature UMAP projections for non-promoters and promoters predictions, respectively, each augmented by density heatmaps that illustrate variations in GC content.

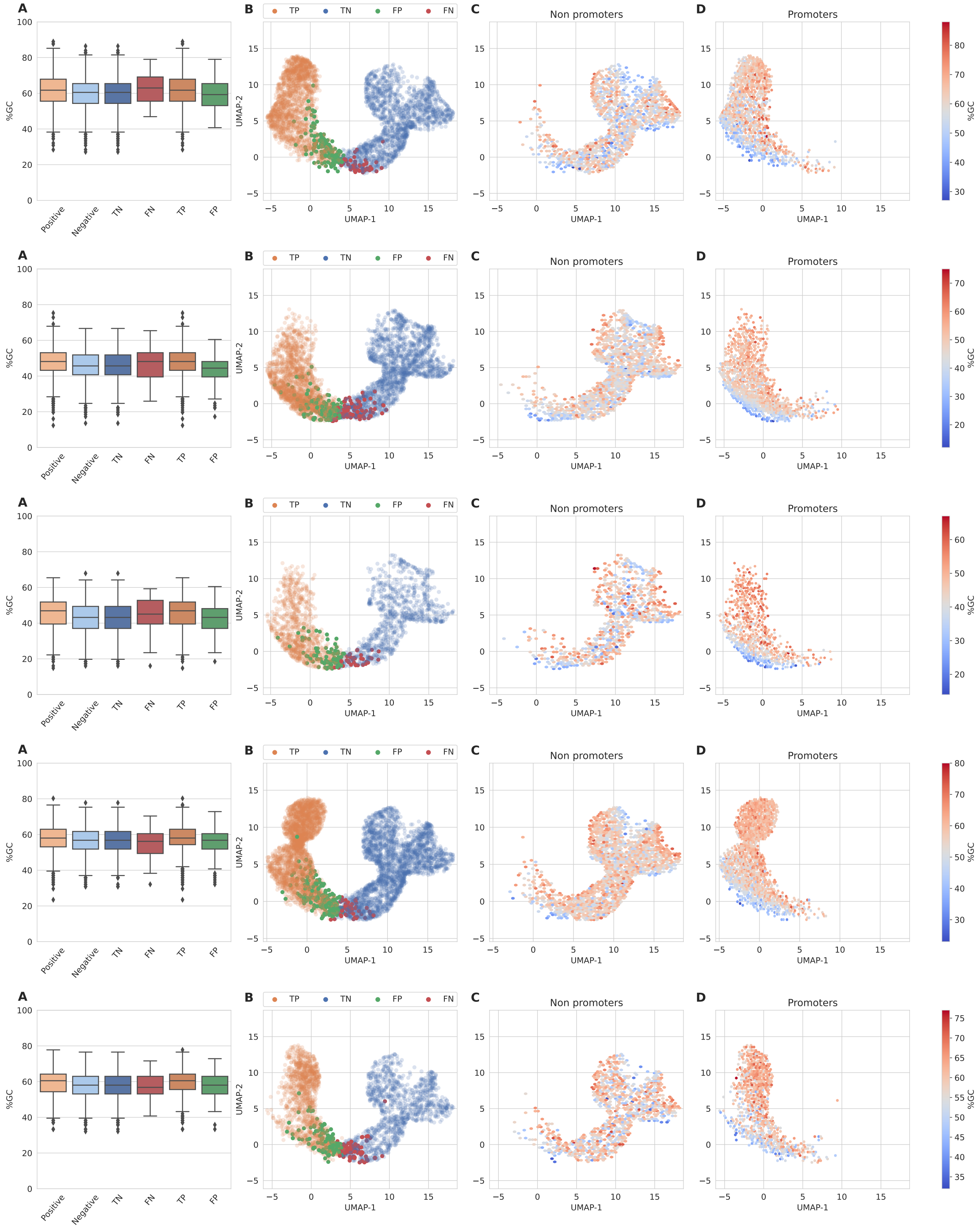

Supplementary Figure S3: **Comparative Analysis of Genomic Sequence Classifications in SRS dataset**. **A**: Boxplots of GC content distribution for actual positive and negative classifications along with predicted categories (TP, TN, FP, FN). **B**: UMAP projections of the `[CLS]` token from DNABERT, based on validation data. **B,D**: feature UMAP projections for non-promoters and promoters predictions, respectively, each augmented by density heatmaps that illustrate variations in GC content.

Supplementary Table S2. Number of promoters and non-promoters sequences obtained in the extraction and before the redundancy filtering phase for each non-promoter origin (CDS & SRS).

	Promoters		Non-promoters		
Specie	Original	Filtered	Original (SRS/CDS)	Filtered (SRS)	Filtered (CDS)
A. baumannii (*Aba*)	1540	1111	3080	3018	2993
A. tumefaciens (*Atu*)	706	698	1412	1400	1389
B. cenocepacia (*Bce*)	10831	9655	21662	21379	19259
B. japonicum (*Bja*)	15933	14760	31866	31691	27445
E. coli (*Eco*)	8616	4920	17232	17110	11338
K. aerogenes (*Kae*)	763	457	1526	1517	1425
P. putida (*Ppu*)	7938	6847	15876	15702	14040
S. flexneri (*Sfl*)	14051	9755	28102	27974	20376
S. meliloti (*Sme*)	17003	14481	34006	33944	27211
X. campestris (*Xca*)	3067	2963	6134	6054	5766
Total	80448	65647	160896	159789	131242

Supplementary Table S3. Total available sequences and mean GC-content on promoters and non-promoter sequences for each dataset, divided by species.

Specie	Total	Prom %GC	Non-prom %GC	
			SRS	CDS
A. baumannii (*Aba*)	2222	32.90	31.72	40.07
A. tumefaciens (*Atu*)	1396	49.04	48.43	59.81
B. japonicum (*Bja*)	19310	59.84	58.33	64.98
B. cenocepacia (*Bce*)	29520	61.41	59.58	67.67
E. coli (*Eco*)	9840	45.27	43.25	51.66
K. aerogenes (*Kae*)	914	44.90	44.76	55.94
P. putida (*Ppu*)	13694	59.24	57.68	62.27
S. flexneri (*Sfl*)	19510	47.36	45.66	51.92
S. meliloti (*Sme*)	28962	58.04	56.31	63.14
X. campestris (*Xca*)	5926	61.03	59.63	65.69
Total	131294	56.05	54.40	61.24

Supplementary Table S4. Overall performance metrics per species for the DNABERT-based model for promoter prediction using the CDS dataset.

Species	Sp	Sn	Pre	Acc	MCC	F_1	ROC AUC
A. baumannii	0.7512	0.9685	0.8083	0.8642	0.7419	0.8811	0.9777
A. tumefaciens	0.8203	0.88	0.8516	0.8525	0.7028	0.8656	0.9126
B. cenocepacia	0.9144	0.8213	0.9068	0.8675	0.7385	0.8619	0.9355
B. japonicum	0.8606	0.7736	0.8465	0.8172	0.6367	0.8084	0.8954
E. coli	0.8584	0.6497	0.817	0.7555	0.5203	0.7238	0.8322
K. aerogenes	0.8043	0.9608	0.8448	0.8866	0.7792	0.8991	0.981
P. putida	0.871	0.6513	0.8361	0.7606	0.5351	0.7323	0.8239
S. flexneri	0.8473	0.8749	0.8548	0.8613	0.7226	0.8647	0.9281
S. meliloti	0.8553	0.6833	0.8297	0.768	0.5459	0.7494	0.8386
X. campestris	0.9240	0.9134	0.9232	0.9187	0.8375	0.9183	0.9707

Supplementary Table S5. Overall performance metrics per species for the DNABERT-based model for promoter prediction using the SRS dataset.

Species	Sp	Sn	Pre	Acc	MCC	F_1	ROC AUC
A. baumannii	0.9268	0.9459	0.9333	0.9368	0.8734	0.9396	0.9779
A. tumefaciens	0.9531	0.9533	0.9597	0.9532	0.906	0.9565	0.9855
B. cenocepacia	0.9295	0.9789	0.9337	0.9544	0.9098	0.9558	0.9927
B. japonicum	0.9232	0.9661	0.9259	0.9446	0.89	0.9456	0.988
E. coli	0.9141	0.9605	0.9158	0.937	0.875	0.9376	0.9858
K. aerogenes	0.9565	0.9706	0.9612	0.9639	0.9277	0.9659	0.9907
P. putida	0.9153	0.938	0.9179	0.9267	0.8536	0.9278	0.9823
S. flexneri	0.9288	0.9526	0.9322	0.9409	0.8819	0.9423	0.9845
S. meliloti	0.9312	0.9824	0.9364	0.9572	0.9154	0.9588	0.9931
X. campestris	0.9382	0.9841	0.9409	0.9611	0.9232	0.962	0.9921

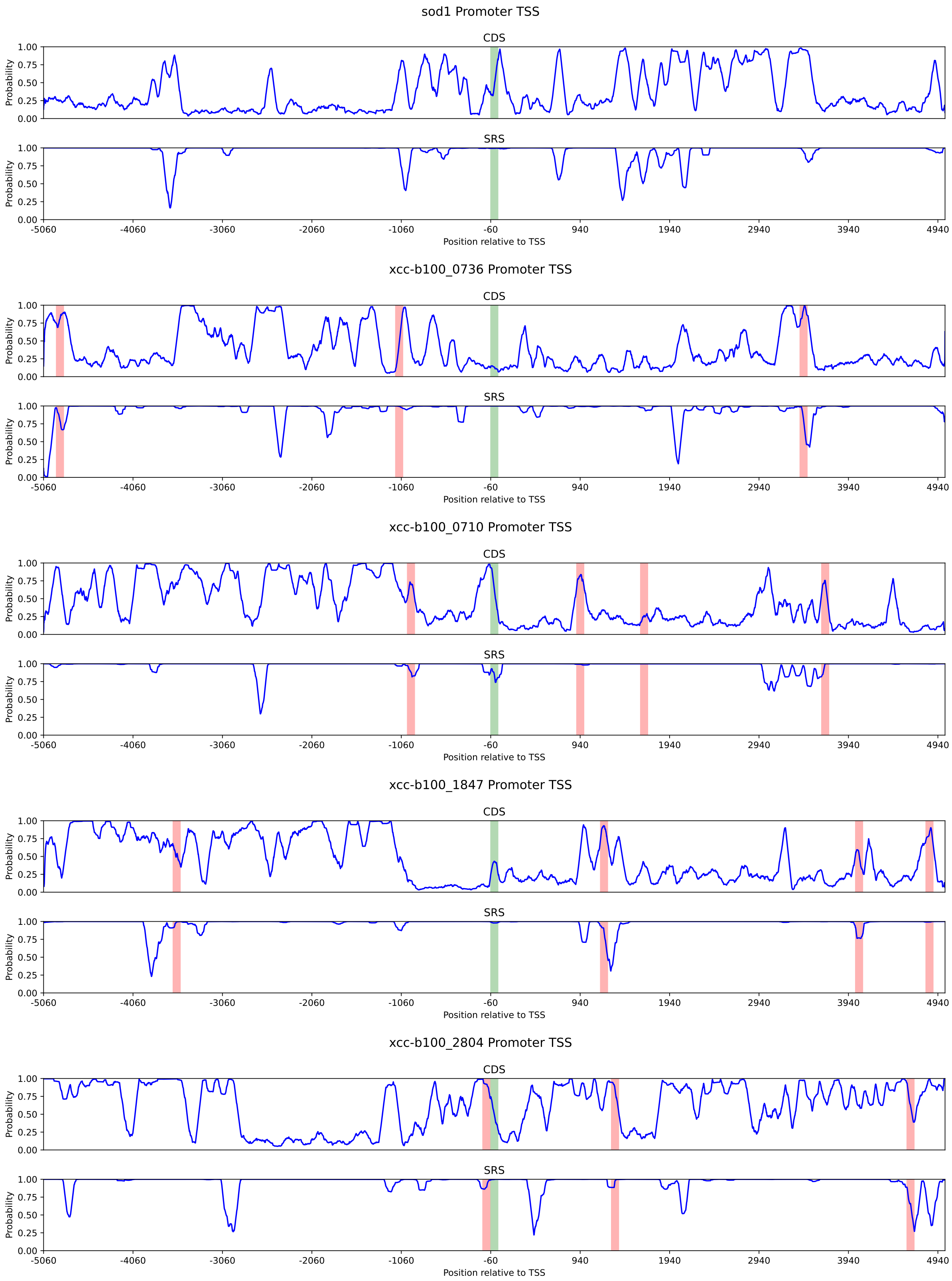

Supplementary Figure S4: Average softmax probabilities per nucleotide predicted by DNABERT for a subset of 10 promoters associated with genes in *X. campestris*, using models trained on CDS (Upper) and SRS (Lower) datasets. Each chart represents a promoter linked to a specific gene. Green highlights indicate validation dataset promoters, while red highlights mark additional promoters included in PPD.

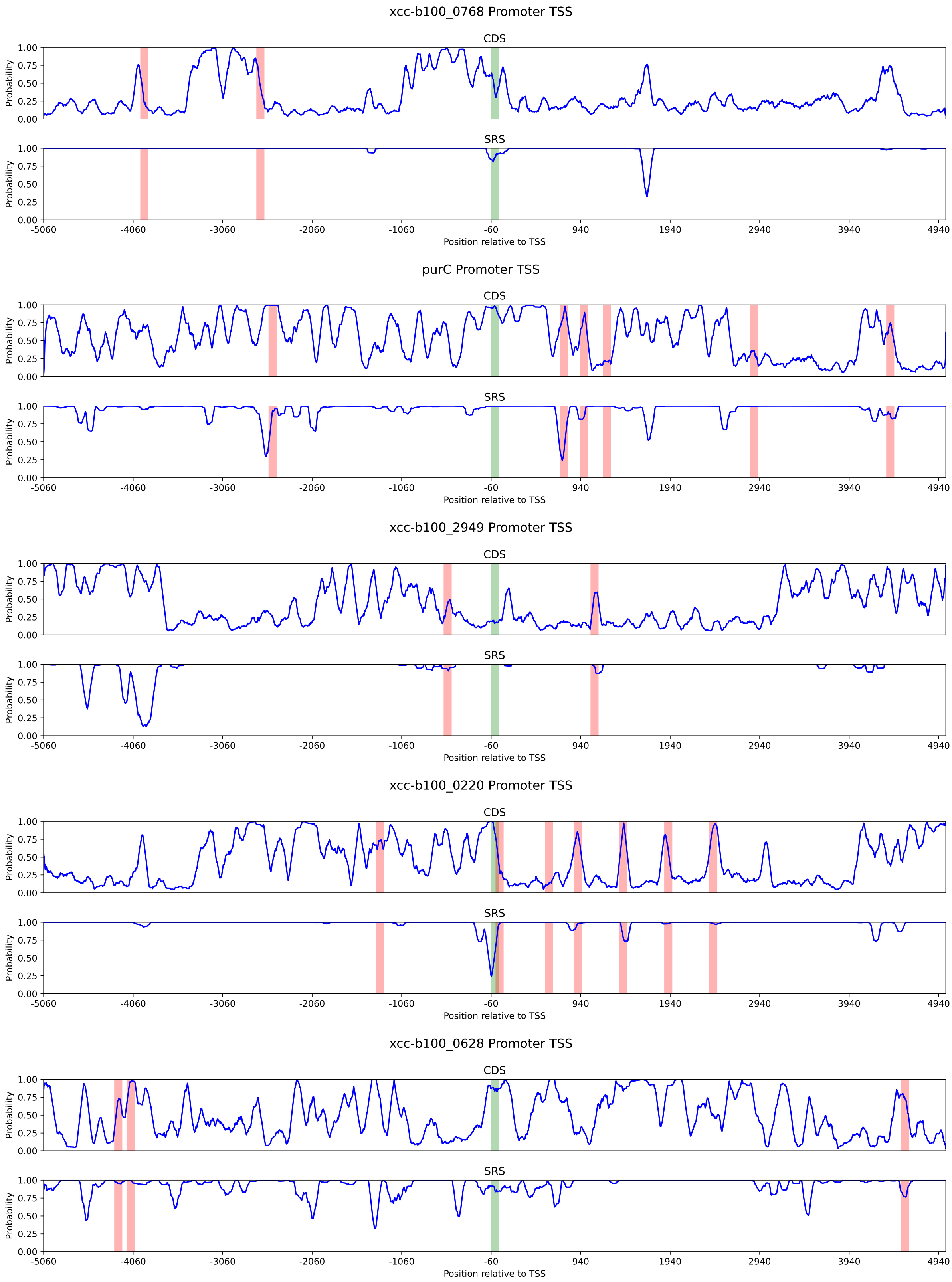

Supplementary Figure S5: Average softmax probabilities per nucleotide predicted by DNABERT for a subset of 10 promoters associated with genes in *X. campestris*, using models trained on CDS (Upper) and SRS (Lower) datasets. Each chart represents a promoter linked to a specific gene. Green highlights indicate validation dataset promoters, while red highlights mark additional promoters included in PPD.
